# Effects of Glucagon-like Peptide-1 Receptor Agonists on Skin Homeostasis and Skin Aging Processes

**DOI:** 10.3390/jcm15082944

**Published:** 2026-04-13

**Authors:** Gabrielė Žaliukaitė, Noura Lebbar

**Affiliations:** 1 Pharmacy and Pharmacology Center, Faculty of Medicine, Vilnius University, LT-01513 Vilnius, Lithuania; 2 Aesthetic Medicine, University of Genoa, 16126 Genoa, Italy; dott.noura.lebbar@gmail.com

**Keywords:** glucagon-like peptide-1 receptor agonists, skin homeostasis, wound healing, skin regeneration, skin aging, dermal white adipose tissue, adipose-derived stem cells

## Abstract

Glucagon-like peptide-1 (GLP-1) is an incretin hormone involved in glucose regulation. Glucagon-like peptide-1 receptor agonists (GLP-1 RAs) are widely used in the treatment of type 2 diabetes mellitus and obesity, as well as in cardiovascular risk reduction. Recent evidence suggests that GLP-1 receptors are expressed in cutaneous tissues; however, their role in skin homeostasis and aging remains insufficiently clarified. This review summarizes recent experimental and clinical studies examining the effects of GLP-1 receptor agonists on skin homeostasis, wound healing, regeneration, and aging processes. Experimental data indicate that GLP-1 RAs may promote wound healing through modulation of inflammatory pathways, enhancement of keratinocyte migration, improved microvascular perfusion, and support of fibroblast function. Antioxidant and cytoprotective mechanisms have also been described. Conversely, rapid weight loss associated with GLP-1 RA therapy has been linked to structural facial changes, including reduction in dermal white adipose tissue and decreased collagen synthesis, which may clinically resemble accelerated skin aging. Mechanistic findings suggest heterogeneous and pathway-dependent effects. Overall, the impact of GLP-1 receptor agonists on skin biology appears multifaceted, and further well-designed clinical studies are required to determine their precise dermatological implications.

## 1. Introduction

Glucagon-like peptide-1 (GLP-1) is an incretin hormone secreted by intestinal endocrine cells in response to food intake [1,2,3,4,5]. GLP-1 binds to specific receptors expressed in various tissues throughout the body and plays a key role in the regulation of glucose homeostasis [1,5,6]. The therapeutic potential of GLP-1 led to the development of glucagon-like peptide-1 receptor agonists (GLP-1 RAs), which mimic the effects of endogenous GLP-1 [7]. GLP-1 RAs are structurally modified peptides that are resistant to degradation by dipeptidyl peptidase-4 (DPP-4), resulting in a prolonged biological half-life compared with native GLP-1 [8]. These agents regulate blood glucose levels by stimulating glucose-dependent insulin secretion and suppressing glucagon release [9,10]. Clinical trials have confirmed the efficacy of GLP-1 RAs in the treatment of type 2 diabetes mellitus, obesity, and in reducing cardiovascular risk [2,3,4,5,6,7,9]. Treatment with GLP-1 RAs has been shown to induce substantial weight loss, reaching up to 15% over one to two years [11]. Beyond their metabolic effects, an increasing body of experimental and clinical evidence suggests that GLP-1 RAs may exert immunomodulatory and anti-inflammatory effects [4,5]. In addition, potential effects on cellular energy metabolism, autophagy-related processes, and neuroprotective signaling pathways have been described; however, the clinical relevance of these mechanisms has not yet been fully elucidated [5].

Several glucagon-like peptide-1 receptor agonists with high biological activity and a favorable safety profile are currently used in clinical practice. The main pharmacological characteristics of these agents and the GLP-1 receptor agonists approved for clinical use are summarized in Table 1 [4].

Combination therapies are also used in clinical practice, including fixed-dose combinations of liraglutide with insulin degludec and lixisenatide with insulin glargine [1]. In addition, dual and triple agonists related to GLP-1 signaling have been actively developed in recent years. The concept underlying their development is based on the simultaneous targeting of multiple key metabolic pathways in order to achieve more effective glycemic control, greater weight reduction, and overall improvement of metabolic status [8].

The use of GLP-1 receptor agonists is rapidly increasing worldwide. Since 2020, prescriptions of GLP-1 RAs in the United States for patients without type 2 diabetes mellitus have increased by approximately 700% [12]. According to epidemiological data, approximately 83% of GLP-1 RA prescriptions are issued for the treatment of type 2 diabetes mellitus, while the remaining proportion is prescribed for obesity management [13]. Although detailed, publicly available data on the extent of GLP-1 RA use in Lithuania are currently lacking, an increasing prescription of these agents is also observed in clinical practice, reflecting broader international trends.

With the increasing use of GLP-1 receptor agonists, growing attention has been directed toward their effects not only on metabolic processes but also on other organs and physiological systems, including the skin. As GLP-1 receptors are expressed in various tissues, including the skin, it is assumed that GLP-1 RA therapy may influence biological processes occurring within the skin [4,9,11]. However, the role of GLP-1 receptors in skin homeostasis has not yet been fully elucidated [1], and therefore, the effects of these agents on skin aging and regenerative processes remain a subject of ongoing debate.

In recent years, with the increasing use of GLP-1 receptor agonists, the so-called “Ozempic face” has been increasingly discussed in the literature and clinical practice. This term refers to weight loss-associated changes in facial appearance that resemble accelerated aging [3,6,9,11,14,15,16,17]. The term was first introduced by dermatologist Paul Jarrold Frank to describe facial changes observed in patients using semaglutide for weight reduction purposes [17]. This phenomenon is most commonly associated with a reduction in facial subcutaneous adipose tissue, which may lead to visible signs of skin laxity, accentuated wrinkles, and alterations in facial contours [3,9,11,14,17,18,19]. However, it remains unclear whether these changes represent an inevitable consequence of GLP-1 RA therapy, are primarily related to rapid and uneven loss of adipose tissue, or may also be influenced by other biological mechanisms that have not yet been sufficiently investigated [9].

Although GLP-1 receptor agonists are widely used in the treatment of type 2 diabetes mellitus and obesity, their effects on skin homeostasis, regeneration, and aging processes remain insufficiently studied. Therefore, it is justified to review the most recent experimental and clinical studies examining the effects of GLP-1 RAs on biological processes occurring within the skin.

The aim of this review was to systematically evaluate scientific studies investigating the effects of glucagon-like peptide-1 receptor agonists on skin homeostasis and skin aging processes.

## 2. Methodology

### 2.1. Objective

This article presents a structured narrative review of scientific studies investigating the effects of glucagon-like peptide-1 receptor agonists (GLP-1 RAs) on skin homeostasis, regenerative processes, and skin aging. The aim of this review is to synthesize current evidence on the biological, clinical, and mechanistic effects of GLP-1 receptor agonists on skin and subcutaneous tissues, including both regenerative and aging-related processes.

### 2.2. Literature Search and Study Selection

The literature search was conducted to identify relevant studies evaluating the effects of GLP-1 receptor agonists on skin biology. Searches were performed in the PubMed (MEDLINE), Scopus, and ScienceDirect databases. The search strategy included combinations of the following keywords: “GLP-1 receptor agonist”, “semaglutide”, “liraglutide”, “skin”, “wound healing”, “skin aging”, “dermal white adipose tissue”, and “adipose-derived stem cells”. Boolean operators (AND, OR) were used to refine the search. The analysis included experimental studies, clinical trials, observational studies, and relevant review articles published between 2017 and 2026 in order to reflect the most recent evidence in this rapidly evolving field. Relevant publications were identified through database searches and subsequently evaluated based on their relevance to the topic. Studies were selected if they provided mechanistic, experimental, or clinical insights into the biological, structural, regenerative, or aesthetic effects of GLP-1 receptor agonists on skin or subcutaneous tissues. The inclusion criteria were as follows: studies investigating the effects of GLP-1 receptor agonists on skin or subcutaneous tissues, including wound healing, skin homeostasis, and skin aging processes. Exclusion criteria included studies focusing exclusively on systemic metabolic outcomes without relevance to skin biology, non-English publications, conference abstracts without full-text availability, studies with insufficient methodological detail, and publications not directly addressing dermatological outcomes. The selected studies were analyzed qualitatively, with emphasis on mechanistic pathways, consistency of findings, and the potential dual effects of GLP-1 receptor agonists on skin physiology. This review represents a narrative synthesis of the currently available evidence.

## 3. Effects of GLP-1 Receptor Agonists on Skin Biology

### 3.1. Effects of GLP-1 Receptor Agonists on Skin Regeneration and Wound Healing

The skin is the largest organ of the human body and performs essential protective, sensory, thermoregulatory, and metabolic functions [20,21,22]. Skin homeostasis is defined as a dynamic process that ensures the stable maintenance of skin structure and function despite external environmental influences. It encompasses the preservation of epidermal barrier integrity, regulation of transepidermal water loss, protection against microorganisms, cellular renewal, and effective wound healing [20]. Maintenance of skin homeostasis is essential for normal barrier function, and any disruption of this balance may lead to increased susceptibility to infections, inflammatory processes, and delayed tissue repair [21]. For this reason, systemic factors capable of modulating inflammation, cellular metabolism, or regenerative processes may have a significant impact on skin homeostasis.

Skin homeostasis is closely linked to the processes of cutaneous wound healing. Wound healing proceeds through several interrelated phases—namely, the inflammatory, proliferative, and remodeling phases—during which various cell types and signaling mediators act in a coordinated manner. Throughout all of these phases, fibroblasts play a central role by regulating extracellular matrix formation through the synthesis of collagen, cytokines, matrix metalloproteinases (MMPs), and their tissue inhibitors (TIMPs). A balanced MMP–TIMP ratio is essential for effective wound healing, whereas increased MMP activity is associated with delayed tissue repair. Prolongation of the inflammatory phase leads to increased fibroblast activity and enhanced secretion of transforming growth factor beta (TGFβ1, TGFβ2) and insulin-like growth factor 1 (IGF-1). In patients with type 2 diabetes mellitus and in chronic cutaneous wounds, elevated levels of MMP-9 and reduced TIMP expression are frequently observed, which negatively affect the wound healing process [22].

Scientific evidence suggests that glucagon-like peptide-1 receptor agonists may influence cutaneous wound healing processes, which are closely associated with the maintenance of skin homeostasis [1,10,11,23]. Experimental data indicate that GLP-1 RAs may modulate signaling pathways involved in fibrosis formation and wound healing, thereby contributing to tissue regeneration processes [6]. Studies investigating the effects of GLP-1 RAs on diabetic foot ulcers, both in clinical settings and in vivo models, have demonstrated accelerated wound healing compared with control groups [24]. In addition, GLP-1 RAs have been reported to suppress inflammatory signaling and reduce fibrotic processes in various tissues, including the skin, which may be relevant for the maintenance of normal wound healing [25].

Experimental studies indicate that glucagon-like peptide-1 receptor agonists can significantly accelerate cutaneous wound healing in both in vitro and in vivo models. Wolak M, Staszewska T et al. [26] demonstrated that GLP-1 RAs reduce MMP-9 levels and the MMP-9/TIMP ratio, which is associated with more favorable conditions for tissue regeneration. In animal models, liraglutide has been shown to promote keratinocyte migration and accelerate wound healing through activation of the phosphatidylinositol 3-kinase/protein kinase B (PI3K/Akt) signaling pathway [27]. Similar findings were reported by Nagae K, Uchi H et al. [23], whose experimental studies in mice demonstrated that liraglutide enhances keratinocyte migratory activity, a critical step in epidermal regeneration and wound closure. Activation of GLP-1R has also been associated with a reduced inflammatory response, enhanced angiogenesis, and modulation of transforming growth factor beta (TGF-β) and MMP signaling molecules, thereby creating a more favorable environment for tissue repair [23]. In addition, other studies suggest that GLP-1 may improve wound healing by enhancing angiogenesis and fibroblast activity, which are essential for extracellular matrix formation and tissue repair. This effect is thought to be mediated, at least in part, by modulation of growth factor-related signaling pathways and reduction in local inflammatory responses, thereby creating a more favorable microenvironment for tissue regeneration [10,23].

Activation of the GLP-1 receptor has been associated with the activation of growth factor-related signaling pathways, which may be relevant to cutaneous wound healing and tissue regeneration processes [27,28]. Experimental data suggest that GLP-1 receptor agonists may indirectly influence pathways involved in tissue regeneration and extracellular matrix remodeling; however, their direct effects on collagen synthesis and skin structure remain incompletely understood and appear to be context-dependent [29]. In addition, the effects of GLP-1 RAs have been linked to modulation of microvascular endothelial function in the skin and subcutaneous tissue [9]. Studies indicate that these agents may directly and indirectly affect arterioles and capillaries, thereby increasing microvascular perfusion [30]. Improved blood flow may create more favorable conditions for tissue oxygenation and nutrient delivery, which could potentially contribute to more effective wound healing and skin regeneration [9,31].

Anastasiou IA, Tentolouris A et al. [7] demonstrated that, under conditions of oxidative stress, semaglutide significantly increased cell viability and adenosine triphosphate (ATP) production (*p* < 0.001), while concurrently reducing levels of apoptosis and reactive oxygen species (ROS). This effect was associated with accelerated fibroblast migration and wound healing, with experimental models achieving near-complete tissue restoration. Gene expression analysis revealed upregulated expression of antioxidant- and extracellular matrix (ECM)-related genes, alongside downregulated expression of pro-inflammatory cytokines and matrix metalloproteinases (MMPs). These findings suggest that semaglutide exerts pronounced antioxidant and cytoprotective effects in skin fibroblasts, thereby creating favorable conditions for tissue regeneration and wound healing [7]. These effects may be partly mediated by upregulation of antioxidant defense-related genes and activation of cellular pathways involved in oxidative stress response, such as the nuclear factor erythroid 2-related factor 2 (Nrf2) signaling pathway, which plays a key role in cellular protection against oxidative stress.

According to some studies, glucagon-like peptide-1 receptor agonists may exhibit synergistic effects when applied locally in combination with other biologically active agents, such as zinc oxide nanoparticles or adipose-derived stem cells, thereby further improving cutaneous wound healing outcomes [32]. These findings support consideration of a potential role for GLP-1 RAs in the prevention and treatment strategies of diabetic wounds [11].

Overall, it can be concluded that GLP-1 receptor agonists may exert beneficial effects on cutaneous wound healing and tissue regeneration, primarily through improved glycemic control, modulation of inflammatory responses, and support of keratinocyte and fibroblast function. These mechanisms suggest that GLP-1 RAs may contribute to the maintenance of skin homeostasis. However, at present, the majority of available evidence is derived from experimental in vitro and animal model studies, and robust clinical data remain limited.

### 3.2. Effects of GLP-1 Receptor Agonists on Skin Aging Processes

Skin aging is a complex biological process characterized by structural and functional changes in both the epidermis and dermis, leading to skin thinning, dryness, reduced elasticity, and the formation of wrinkles [9]. Aging skin exhibits decreased cellular proliferative activity, impaired barrier function, and reduced synthesis of collagen and elastin. Due to a limited capacity for renewal, cells gradually lose their proliferative potential and become more susceptible to apoptosis [33].

Two main types of skin aging are distinguished: intrinsic aging, which is primarily determined by genetic and hormonal factors, and extrinsic aging, which is associated with environmental influences, including ultraviolet radiation, pollution, and lifestyle-related factors. Both types of aging are linked to dysregulation of neuroimmune and endocrine mechanisms, which may disrupt skin homeostasis [21].

One of the principal pathogenic factors in skin aging is oxidative stress, which arises from reduced activity of antioxidant defense systems and increased production of reactive oxygen species (ROS). Excess ROS leads to DNA damage, oxidation of membrane lipids, and disruption of intracellular signaling. In addition, oxidative stress may activate the mitogen-activated protein kinase (MAPK) signaling pathway, which suppresses procollagen synthesis, as well as nuclear factor κB (NF-κB), which promotes the production of pro-inflammatory cytokines and adversely affects collagen metabolism [9].

**Figure 1:**
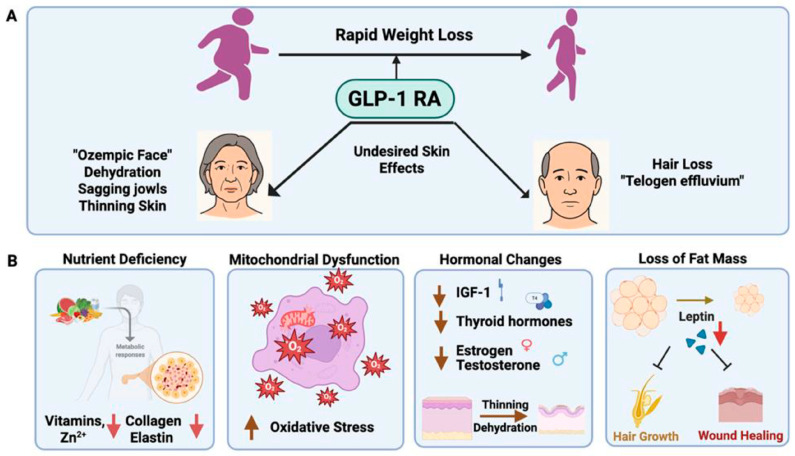
Potential mechanisms underlying adverse cutaneous effects associated with GLP-1 receptor agonist use. (**A**) GLP-1 RA-induced rapid weight loss may be associated with adverse dermatological changes, including reduction in facial volume (“Ozempic face”), skin dehydration, sagging of subcutaneous tissues, skin thinning, as well as hair loss (telogen effluvium). (**B**) Several interrelated mechanisms may contribute to the pathogenesis of these changes: nutrient deficiencies (vitamins, zinc) and reduced synthesis of collagen and elastin, mitochondrial dysfunction and increased oxidative stress, hormonal alterations, including reduced levels of insulin-like growth factor 1 (IGF-1), thyroid hormones, estrogen, and testosterone, leading to skin thinning and dehydration, reduction in adipose tissue mass, accompanied by decreased leptin levels, potentially impairing skin structure, wound healing, and hair growth. Source: Adapted from Cao K [15].

The use of glucagon-like peptide-1 receptor agonists has been associated with the so-called “Ozempic face” phenomenon, which is characterized by signs of accelerated facial skin aging [3,6,9,11,14,15,16,17]. One of the main proposed mechanisms underlying this phenomenon is rapid loss of subcutaneous adipose tissue, which may alter facial proportions, reduce soft tissue volume, and create the visual appearance of aged skin [3,9,14,15,16,17,19]. The pathophysiological mechanisms of facial skin changes potentially associated with GLP-1 RA use are schematically illustrated in Figure 1. 

According to the literature, one of the most important proposed mechanisms underlying the pathogenesis of the “Ozempic face” phenomenon is the reduction in dermal white adipose tissue (DWAT), which is considered a significant regulator of skin structure, elasticity, and aging processes [6]. Rosenbloom AJ, Gasbeck TP et al. [6] report that loss of DWAT may contribute to skin thinning, reduced soft tissue volume, and the development of more pronounced signs of aging. These changes are thought not to be exclusively related to the pharmacological effects of GLP-1 receptor agonists, but rather to reflect the consequences of rapid weight loss and potential nutritional deficiencies. Similar dermatological changes have also been described in patients following bariatric surgical procedures, which are characterized by abrupt loss of adipose tissue [9,19].

Review studies indicate that in patients receiving GLP-1 receptor agonists, signs of facial skin aging may appear earlier than would be expected during the natural aging process [14]. These changes may negatively affect psychosocial well-being, self-esteem, and health-related quality of life, particularly in individuals who are sensitive to changes in physical appearance [15,16].

DeVore S, Reimer H et al. [14], based on experimental and clinical data, suggest that long-term semaglutide use may be associated with reduced skin elasticity. Elastin is one of the principal structural proteins of the dermis, responsible for skin flexibility and the ability to return to its original shape, and its content naturally declines with aging. A reduction in elastin, together with loss of subcutaneous adipose tissue, may contribute to skin laxity and the development of more pronounced signs of aging. The authors also discuss a potential effect of GLP-1 receptor agonists on adipose-derived stem cells (ADSCs), which play a crucial role in dermal regeneration and tissue repair. It is hypothesized that GLP-1 RAs may modulate ADSC proliferation and differentiation processes, which could in turn influence fibroblast activity and extracellular matrix renewal [14]. These mechanisms support the consideration that the effects of GLP-1 RAs on subcutaneous adipose tissue cells and dermal structural components may represent one of the contributing factors to the development of accelerated facial skin aging. Similar hypotheses are supported by other authors, who point to a potential influence of GLP-1 RAs on the metabolic activity and proliferative capacity of cells within the subcutaneous adipose layer [9].

Ridha Z, Fabi SG et al. [34] investigated the potential effects of GLP-1 receptor agonists on facial tissues, including dermal white adipose tissue (DWAT), adipose-derived stem cells (ADSCs), and facial muscles. The authors demonstrated that GLP-1 receptors are expressed within the DWAT layer, which is considered an important regulator of skin structure and aging processes. A reduction in DWAT volume has been associated with skin aging, as it is accompanied by a decreased number of collagen-synthesizing cells and increased collagen degradation related to elevated matrix metalloproteinase-1 (MMP-1) activity.

ADSCs are mesenchymal-derived cells characterized by their capacity for differentiation and participation in tissue regeneration, including collagen synthesis and maintenance of the extracellular matrix. The authors report that activation of GLP-1 receptors on the surface of ADSCs may modulate their proliferative and differentiation processes. This may, in turn, influence fibroblast migration and their ability to synthesize extracellular matrix proteins that are essential for maintaining normal dermal structure [34]. These findings suggest that the effects of GLP-1 RAs on DWAT and ADSC function may represent one of the mechanisms contributing to the development of accelerated facial skin aging.

**Figure 2:**
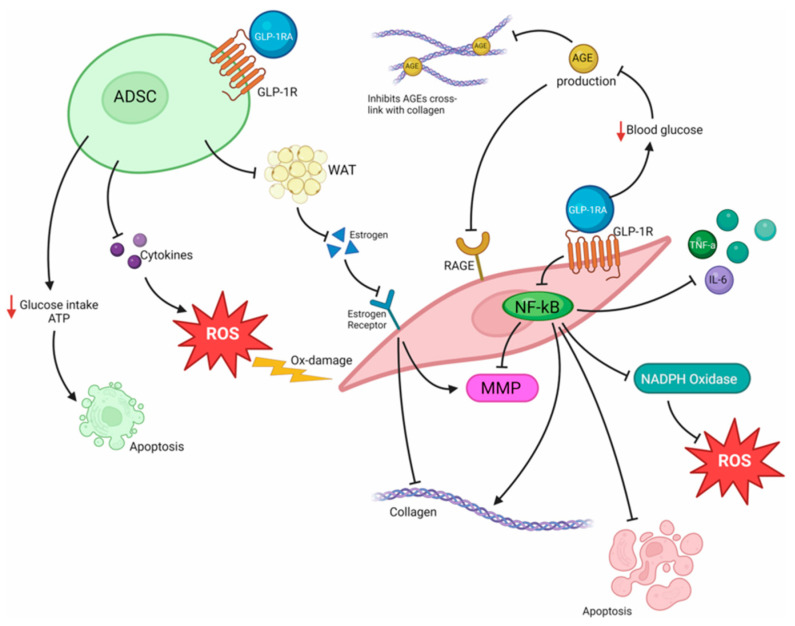
Potential mechanisms by which GLP-1 receptor agonists may influence skin aging processes. The figure illustrates mechanisms predominantly derived from experimental in vitro and in vivo studies; their clinical relevance to human skin aging has not yet been definitively established. ADSCs (adipose-derived stem cells) may be affected by GLP-1 receptor activation, leading to alterations in glucose uptake and ATP production, modulation of cytokine secretion, increased susceptibility to oxidative stress, and induction of apoptotic pathways. GLP-1 receptor activation may also influence dermal white adipose tissue (DWAT), indirectly affecting local estrogen production. Reduced estrogen signaling may impair fibroblast function and collagen synthesis. In fibroblasts, GLP-1 receptor-related signaling pathways may interact with advanced glycation end-products (AGEs) and their receptor (RAGE), influencing NF-κB activation, pro-inflammatory cytokine release (e.g., TNF-α, IL-6), matrix metalloproteinase (MMP) expression, and NADPH oxidase-mediated reactive oxygen species (ROS) production. These processes may contribute to increased collagen degradation, oxidative damage, and apoptosis, thereby promoting structural and functional features of skin aging. Abbreviations: ADSC—adipose-derived stem cells; ROS—reactive oxygen species; AGE—advanced glycation end-products; RAGE—receptor for advanced glycation end-products; MMP—matrix metalloproteinases; NADPH oxidase—enzyme complex involved in cellular ROS generation; NF-κB—nuclear factor kappa-light-chain-enhancer of activated B cells, a transcription factor regulating genes involved in inflammation, immune responses, and cell survival. Source: Adapted from Paschou IA, Sali E et al. [9].

Paschou IA, Sali E et al. [9] review the potential mechanisms through which GLP-1 receptor agonists may influence skin aging processes, which are schematically illustrated in Figure 2. According to the authors, GLP-1 RAs may affect adipose-derived stem cells (ADSCs) and fibroblasts via GLP-1 receptors expressed on their cell surfaces. Activation of GLP-1R in ADSCs may modulate their metabolic and secretory activity, including the expression of cytokines and growth factors that are essential for maintaining normal fibroblast function. Reduced availability of these signaling molecules may render fibroblasts more susceptible to oxidative stress, leading to increased accumulation of reactive oxygen species (ROS) and oxidative damage. Furthermore, the effects of GLP-1 RAs on glucose metabolism in ADSCs may result in decreased glucose uptake and adenosine triphosphate (ATP) production, which under unfavorable conditions may promote cellular dysfunction and apoptotic processes. The authors also indicate that GLP-1R activation within dermal white adipose tissue (DWAT) may indirectly affect local estrogen synthesis. As estrogens stimulate fibroblast activity and collagen production, a reduction in estrogen levels may contribute to decreased collagen synthesis and accelerated manifestations of skin aging [9].

Glucagon-like peptide-1 receptor agonists (GLP-1 RAs) may influence skin aging processes through several interrelated biological pathways. Activation of GLP-1 receptors (GLP-1R) in adipose-derived stem cells (ADSCs) and fibroblasts may modulate ADSC proliferation and differentiation, reduce the secretion of cytokines and growth factors essential for maintaining fibroblast function, and affect the ability of fibroblasts to synthesize collagen. Reduced glucose uptake and adenosine triphosphate (ATP) production may lead to increased oxidative stress, accumulation of reactive oxygen species (ROS), and activation of apoptotic pathways. GLP-1 RAs may also affect dermal white adipose tissue (DWAT), in which GLP-1 receptors are expressed. Activation of GLP-1R within this tissue may indirectly influence local estrogen synthesis, and reduced estrogen signaling has been associated with decreased collagen production in the dermis. In addition, improved glycemic control reduces the formation of advanced glycation end-products (AGEs) and their interaction with receptors for AGEs (RAGE); however, inflammatory cytokine and matrix metalloproteinase (MMP) expression may simultaneously be modulated via NF-κB and NADPH oxidase signaling pathways, thereby influencing extracellular matrix degradation. Collectively, these mechanisms may contribute to alterations in dermal structure, reduced skin elasticity, and the development of clinical signs of skin aging.

Conversely, Paschou IA, Sali E et al. [9] indicate that GLP-1 receptor agonists may modulate skin aging processes through the metabolism of advanced glycation end-products (AGEs). GLP-1 RAs may reduce AGE formation and inhibit their binding to collagen, thereby contributing to preservation of extracellular matrix structure. In addition, GLP-1 RAs may suppress downstream signaling following activation of the receptor for advanced glycation end-products (RAGE), resulting in reduced activation of the nuclear transcription factor NF-κB. Consequently, production of pro-inflammatory cytokines is decreased, NADPH oxidase activation and reactive oxygen species (ROS) generation are attenuated, fibroblast apoptosis and matrix metalloproteinase (MMP) activity are reduced, and collagen homeostasis within the skin may be preserved.

According to Paschou IA, Sali E et al. [9], two distinct mechanisms through which GLP-1 receptor agonists may influence skin aging processes can be identified. Interaction of GLP-1 RAs with adipose-derived stem cells (ADSCs) has been associated with impaired skin homeostasis and a potential acceleration of skin aging processes. In contrast, the ability of GLP-1 RAs to reduce the formation of advanced glycation end-products (AGEs) and inhibit activation of their receptor (RAGE) is considered a skin-protective mechanism that may contribute to attenuation of skin aging. Thus, the effects of GLP-1 RAs on skin aging are multifaceted and depend on the predominance of specific molecular pathways. The interplay between these mechanisms suggests that the biological impact of these agents on skin aging processes may be either detrimental or protective.

At present, there is a lack of clinical data that would allow for a comprehensive explanation of the mechanisms through which GLP-1 receptor agonists influence skin aging processes. Most of the available evidence is derived from experimental in vitro and in vivo studies or mechanistic laboratory investigations, whereas well-designed clinical studies in humans remain limited [5]. Consequently, it remains unclear whether GLP-1 RAs directly contribute to the so-called “facial aging” phenomenon. The literature emphasizes the need for further clinical research to determine whether these changes represent a direct biochemical effect of GLP-1 RAs on skin tissues or are predominantly related to rapid weight loss and the resulting morphological alterations of facial structures [6,14].

Signs of the so-called “Ozempic face” associated with the use of GLP-1 receptor agonists have led to an increasing demand for personalized aesthetic interventions [3,16]. In clinical practice, approaches such as dermal fillers, biostimulatory agents, and skin-tightening technologies may help restore lost facial volume, improve skin elasticity, and enhance overall skin structure [3]. The importance of general skin care measures is also emphasized, including adequate hydration, balanced nutrition, and the use of targeted topical products with ceramides, collagen-stimulating, or antioxidant properties [15]. Informing patients about potential skin changes associated with GLP-1 RA therapy allows for early identification of undesirable effects and promotes collaboration with healthcare professionals, thereby enabling continuation of treatment while minimizing potential negative impacts on facial skin condition [15].

## 4. Discussion

The expanding use of GLP-1 receptor agonists has shifted attention beyond metabolic control toward their systemic biological implications, including potential effects on skin physiology. The present analysis suggests that the dermatological impact of GLP-1 RAs cannot be interpreted in a linear or uniformly beneficial or harmful manner. Instead, their effects appear to depend on the interaction between metabolic improvement, tissue-specific receptor signaling, and structural adaptations secondary to weight reduction.

One of the central complexities emerging from the literature is the apparent divergence between experimental regenerative findings and clinical observations of structural facial changes. Mechanistic studies indicate enhanced cellular viability, improved modulation of inflammation pathways, and support of regenerative processes following GLP-1 receptor activation. However, real-world aesthetic concerns—particularly facial volume loss and altered skin elasticity—are largely associated with rapid adipose tissue reduction rather than direct molecular injury to dermal structures. This distinction is critical, as it reframes the discussion from drug-induced dermal damage to systemic body composition remodeling with visible cutaneous consequences.

Furthermore, the role of dermal white adipose tissue (DWAT) and adipose-derived stem cells (ADSCs) highlights the dualistic biological context in which GLP-1 RAs operate. While metabolic improvement may reduce glycation-related dermal degradation and inflammatory burden, alterations in adipocyte signaling and subcutaneous tissue dynamics may indirectly influence extracellular matrix stability. Whether these changes represent transient adaptive remodeling or sustained structural modification remains insufficiently clarified.

Another important consideration is the heterogeneity of the available evidence. Most mechanistic data originate from in vitro or animal models, whereas clinical dermatological endpoints remain underexplored. In addition, aesthetic observations frequently derive from case-based or observational contexts without standardized measurement of dermal thickness, collagen density, or microvascular parameters. This limitation reduces the ability to establish causality and underscores the need for objective dermatological outcome measures in future prospective studies.

From a clinical perspective, these findings emphasize the importance of contextual interpretation. The metabolic and cardiovascular benefits of GLP-1 receptor agonists are well established and often substantial. Dermatological and aesthetic changes, when present, should therefore be considered within a broader therapeutic risk–benefit framework. Early patient counseling and interdisciplinary collaboration between metabolic and dermatological specialists may help optimize both systemic and aesthetic outcomes.

Ultimately, the current body of evidence suggests that GLP-1 receptor agonists influence skin biology through interconnected metabolic, inflammatory, and structural pathways. However, whether observed cutaneous changes represent direct pharmacological effects or indirect consequences of rapid systemic adaptation remains an open question. Clarifying this distinction will require well-designed longitudinal studies incorporating objective dermatological measurements and controlled evaluation of weight loss kinetics.

## 5. Conclusions

Glucagon-like peptide-1 receptor agonists (GLP-1 RAs) exhibit pleiotropic effects that extend beyond the regulation of metabolic processes and include potential roles in the maintenance of skin homeostasis. Experimental evidence suggests that GLP-1 RAs may promote cutaneous wound healing and tissue regeneration through improved glycemic control, modulation of inflammatory responses, and support of keratinocyte and fibroblast function. At the same time, the effects of GLP-1 RAs on skin aging processes remain heterogeneous. While certain mechanisms, particularly those related to suppression of adipose-derived stem cell activity and reduction in subcutaneous adipose tissue, may be associated with accelerated skin aging, other data indicate potential protective effects mediated through reduction in advanced glycation end-products and attenuation of inflammatory signaling pathways. As the majority of currently available evidence is derived from in vitro studies and animal models, well-designed clinical studies in humans are still limited. The dualistic nature of GLP-1 receptor agonist-related mechanisms suggests that their dermatological impact cannot be interpreted in a unidirectional manner. The apparent discrepancy between regenerative experimental findings and clinically observed structural changes emphasizes the need to distinguish direct pharmacological effects from secondary consequences of rapid weight loss. Therefore, further research is required to more precisely evaluate the impact of GLP-1 RAs on skin homeostasis and aging processes.

## Figures and Tables

**Table 1:** Glucagon-like peptide-1 receptor agonists and related agents used in clinical practice. Trade names and approved indications are based on the cited source and may vary across countries and regulatory authorities. Source: Paschou IA, Sali E et al. [4].

Generic Name	Trade Name	Indication
Liraglutide	Victoza	Type 2 diabetes mellitus
Liraglutide	Saxenda	Obesity
Semaglutide	Ozempic	Type 2 diabetes mellitus
Semaglutide	Wegovy	Obesity
Semaglutide	Rybelsus	Type 2 diabetes mellitus
Exenatide	Byetta	Type 2 diabetes mellitus
Exenatide	Bydureon BCise	Type 2 diabetes mellitus
Dulaglutide	Trylicity	Type 2 diabetes mellitus
Lixisenatide	Adlyxin	Type 2 diabetes mellitus
Tirzepatide *	Mounjaro	Type 2 diabetes mellitus
Tirzepatide *	Zepbound	Obesity

* Tirzepatide is a dual glucose-dependent insulinotropic polypeptide (GIP) and GLP-1 receptor agonist.

## Data Availability

No new data were created or analyzed in this study. Data sharing is not applicable to this article.
